# External Validation of Robust Radiomic Signature to Predict 2-Year Overall Survival in Non-Small-Cell Lung Cancer

**DOI:** 10.1007/s10278-023-00835-8

**Published:** 2023-09-21

**Authors:** Ashish Kumar Jha, Umeshkumar B. Sherkhane, Sneha Mthun, Vinay Jaiswar, Nilendu Purandare, Kumar Prabhash, Leonard Wee, Venkatesh Rangarajan, Andre Dekker

**Affiliations:** 1 Department of Radiation Oncology (MAASTRO), GROW School for Oncology and Developmental Biology, Maastricht University Medical Center, Maastricht, The Netherlands; 2 Department of Nuclear Medicine, Tata Memorial Hospital, Mumbai, Maharashtra India; 3 Homi Bhabha National Institute, Mumbai, Maharashtra India; 4 Department of Medical Oncology, Tata Memorial Hospital, Mumbai, Maharashtra India

## Abstract

Lung cancer is the second most fatal disease worldwide. In the last few years, radiomics is being explored to develop prediction models for various clinical endpoints in lung cancer. However, the robustness of radiomic features is under question and has been identified as one of the roadblocks in the implementation of a radiomic-based prediction model in the clinic. Many past studies have suggested identifying the robust radiomic feature to develop a prediction model. In our earlier study, we identified robust radiomic features for prediction model development. The objective of this study was to develop and validate the robust radiomic signatures for predicting 2-year overall survival in non-small cell lung cancer (NSCLC). This retrospective study included a cohort of 300 stage I–IV NSCLC patients. Institutional 200 patients’ data were included for training and internal validation and 100 patients’ data from The Cancer Image Archive (TCIA) open-source image repository for external validation. Radiomic features were extracted from the CT images of both cohorts. The feature selection was performed using hierarchical clustering, a Chi-squared test, and recursive feature elimination (RFE). In total, six prediction models were developed using random forest (RF-Model-O, RF-Model-B), gradient boosting (GB-Model-O, GB-Model-B), and support vector(SV-Model-O, SV-Model-B) classifiers to predict 2-year overall survival (OS) on original data as well as balanced data. Model validation was performed using 10-fold cross-validation, internal validation, and external validation. Using a multistep feature selection method, the overall top 10 features were chosen. On internal validation, the two random forest models (RF-Model-O, RF-Model-B) displayed the highest accuracy; their scores on the original and balanced datasets were 0.81 and 0.77 respectively. During external validation, both the random forest models’ accuracy was 0.68. In our study, robust radiomic features showed promising predictive performance to predict 2-year overall survival in NSCLC.

**Supplementary Information:**

The online version contains supplementary material available at 10.1007/s10278-023-00835-8.

## Introduction

Lung cancer is a fatal disease and second-most common cancer worldwide [1]. As per Global Cancer Statistics 2020 (GLOBOCAN 2020), lung cancer is the most common cause of cancer-related death worldwide [2]. Lung cancer alone accounts for 2,093,876 (11.6%) new cases every year and 1,761,007 (18.4%) deaths every year worldwide [2]. Non-small-cell lung cancer (NSCLC) accounts for 85% of lung cancer globally [3]. The prognosis of the disease and survival of the patients grossly depend on the stage of the disease upon diagnosis. Staging of the disease is performed based on the tumor (T), node (N), and metastasis (M) stage (TNM stage) of the disease [4]. TNM staging is often a complex system and depends on imaging, pathology, and clinical assessment. As a consequence, it is sometimes difficult to identify the disease stage very accurately resulting in poor outcomes of treatment.

With the advent of genomic biology and other technical developments, identification of disease sub-group has become more accurate, and survival has improved significantly. For example, gene sequencing by polymerase chain reaction (PCR) assays is a widely used method for the identification of epidermal growth factor receptor (EGFR) mutations in NSCLC patients [3, 5–9]. The diagnosis of the presence of EGFR mutation in NSCLC patients opens the option of targeted therapy using tyrosine kinase inhibitors (TKIs) that improves the overall survival in patients with EGFR mutation [9]. Several driver gene mutations like EGFR, BRAF, KRAS, MET, ALK, and ROS1 were also identified and may be druggable targets. In NSCLC patients, about 32% worldwide and 38% of Asians have EGFR mutations [3–10]. The overall prevalence of EGFR mutation is higher in females (female vs. male: 43.7% vs. 24.0%) [3]. The prevalence of EGFR mutation is also higher in non-smokers (non-smokers vs. past or current smokers: 49.3% vs. 21.5%) [3]. Many other mutations (EGFR, ALK, ROS1, BRAF, NTRK, MET, and RET) in NSCLC patients have resulted in several subgroups. These patients are treated with targeted therapy and personalized treatment [4, 5]. However, these biomarker-guided targeted therapies have improved the survival significantly, but occasionally these treatments fail. In given circumstances, patient selection for these expensive targeted therapies becomes crucial, and radionics-based prediction models may be helpful as shown in various retrospective studies [10].

Radiomics is a new workflow that extracts high-throughput data from medical images called radiomic features. Radiomic features may show a very high correlation with the treatment outcome. Many publications on radiomics have demonstrated the role of radiomic features in the diagnosis and prognosis of the disease in many cancer types [11–17]. Many researchers have demonstrated the role of radiomics in prediction model development and treatment outcome prediction [10]. Aerts, He et al. in their study have demonstrated the potential of radiomic features in the prediction of the overall survival in NSCLC patients [18]. A study by He et al. showed the use of radiomic features in the prediction of progression-free survival in lung cancer [19]. In a similar study, Tunali et al. developed a prediction model to predict local recurrence [20]. Nevertheless, several studies have shown the importance of radiomic signatures in the prediction of various clinical endpoints, and many studies have also raised concerns about the stability of radiomic features [20–22]. The stability of radiomic features is often assessed by measuring similarities in feature values in repeatability (test–retest) and reproducibility studies. It is of utmost importance generalizing the radiomic-based prediction model across the clinic and worldwide. The stability of radiomic features depends on various factors like differences in imaging equipment, imaging parameters or protocols, image reconstruction algorithms, tumor delineation, and pre-processing steps of radiomic feature extraction. The instability of radiomic features has been identified to be a key issue with the generalization of the radiomic-based prediction model [20–22]. Several studies have been performed to identify robust radiomic features among the many features extracted from medical images [20–22]. In our earlier repeatability and reproducibility study, we have identified robust radiomic signature on phantom and clinical cohort [21]. In this study, we aim to develop and validate those robust radiomic signatures for the overall survival prediction in non-small cell lung cancer patients.

## Material and Method

The study was approved by the Institution Ethics Committee (IEC) (IEC-2) of our hospital as a retrospective study. A consent form waiver is provided by the same IEC as an institutional policy. All the data of the patients were kept confidential.

### Patients

#### TMH Dataset

Two hundred patients of non-small cell lung carcinoma (NSCLC) who underwent treatment with a combination of surgery, chemotherapy, and radiotherapy in our hospital from January 2012 to January 2017 were included in this study. The pre-treatment CT or PET/CT scans of these patients was extracted from the hospital PACS and was included. Similarly, clinical data were extracted from the hospital information system (HIS). Patients’ demographic data are shown in Table 1.

**Table 1:** Demographic data of patient population used in this study

Variable		TMH cohort	External validation cohort	*t*-statistics	*p* value
Age (year)	Median	56	71	−10.5	< 0.005
	1st Qu	50	62		
	3rd Qu	64	76		
Sex	Female	65	27	1.7	0.08
	Male	135	73		
Pathology	Adenocarcinoma	161	10	15.8	< 0.005
	Squamous cell carcinoma	32	37		
	Others	7	53		
TNM stage	T1	20	18	0.24	0.81
	T2	98	44		
	T3	48	13		
	T4	34	25		
	N0	77	43	−0.85	0.39
	N1	26	6		
	N2	85	31		
	N3	12	20		
	M0	151	99	5.0	< 0.005
	M1	49	1		
AJCC_stage	IA	9	28	2.7	0.007
	IIA	38	8		
	IIB	32	–		
	III	2	–		
	IIIA	32	24		
	IIIB	11	40		
	IV	76	0		
WHO performance score	0	123	–		
	1	75	–		
	2	2	–		
Treatment	Chemo	78	100		
	Surgery + chemo	122	–		
Overall survival (days)	Median	815.5	416	1.3	0.20
	1st Qu	447.2	172		
	3rd Qu	1219.8	1165		
Survival	< 2 years	110	67		
	> 2 years	90	33		

#### External Validation Set

The Cancer Image Archive (TCIA) open-source data: 100 NSCLC patients with CT images and RT structures (GTV-1) and survival data of NSCLC-radiomics collection were downloaded from the TCIA portal [18, 23]. The CT scans and GTV were used to extract radiomic features.

#### Pre-Processing of Data

Clinical data extracted from the HIS were cleaned and converted into a form amenable to machine learning. CT or PET/CT scans were checked for completeness, and contrast-enhanced CT series of PET/CT or CT studies were selected for this study.

Based on median overall survival (OS) in both the datasets, 2-year OS was selected as a clinical endpoint (Table 1). For both datasets, OS were binarized based on 2-year OS [(OS < 2 years) = 1 and (OS > 2 years) = 0].

### PET/CT Imaging Procedure

#### TMH Dataset

Pre-treatment PET/CT scans were performed using Gemini TF16 or Gemini TF64 PET/CT scanners (Philips Medical Systems, Netherlands). The CT of PET/CT scans were performed after the injection of 60 to 80 ml of non-ionic contrast using the protocol mentioned in Supplementary Table s1. CT images were reconstructed using the filtered back project (FBP) reconstruction algorithm.

#### TCIA External Validation Set

Pre-treatment CT scans were performed using a Gemini CT scanner (Philips Medical Systems, Netherlands). The CT scans were performed after the intravenous injection of 80 ml of non-ionic contrast using the protocol was mentioned in Supplementary Table s1. CT images were reconstructed using the filtered back projection (FBP) reconstruction algorithm.

From both cohorts, CT data were extracted in Digital Imaging and Communications in Medicine (DIOCM) format for radiomic extraction.

#### Radiomic Extraction

##### Internal Dataset

The CT series of PET/CT scans were loaded on Intellispace Discovery Portal (research-only build; Philips Medical System, Eindhoven, The Netherlands) and primary tumor delineation was performed using 3D contouring software by the experienced (more than 15 years) medical physicists and saved as radiotherapy structure (DICOM series: RT structure) by the name of gross tumor volume (GTV). The GTVs were checked and approved by experienced (more than 20 years) nuclear medicine physicians and radiologists. Subsequently, the DICOM images and GTV were transferred to the research computer for radiomic extraction. On a research PC, radiomic features were extracted using in-house developed PyRadGUI software using a combination of Plastimatch [24] and Pyradiomics software [25]. The following pre-processing steps were performed using PyRadGUI software. *Image conversion*: DICOM images and RT structures were converted into NRRD format using the Plastimatch package. *Resampling*: Images were resampled using a 2 × 2 × 2 mm cube isotropic voxel. *Filtering and transformation of image*: Three sets of images were generated applying Laplacian of Gaussian (LoG) filters with sigma values of 1, 2, and 3 mm. We also generated eight sets of wavelet-transformed images using eight combinations of high-pass and low-pass wavelet filters [25]. Finally, a total of 1093 radiomic features were extracted from the 12 imaging sets (1 set of original images, 3 sets of LoG images, and 8 sets of wavelet images) and corresponding GTVs [25].

##### External Validation Set

The TCIA dataset contains CT Image and RT structure (GTV) in DICOM format. We performed the same operation as described in the earlier section, and 1093 radiomic features were extracted for every patient’s data.

#### Data Balancing

Usually, it is assumed that balanced endpoints are more appropriate to train most of the machine learning algorithms for prediction model development [26]. The majority of the time clinical endpoints have imbalanced ratios, which do not meet the assumptions of balanced endpoints and require data balancing. Data balancing was performed using synthetic minority oversampling technique (SMOTE).

#### Prediction Algorithm Used

Several radiomic studies have shown that random forest (RFC), support vector (SVC), and gradient boosting classifier (GBC) algorithms are the most efficient classification algorithms for treatment response and outcome events prediction in radiomics based analysis in several types of cancer (28–30). Hence, in this study, we have used RFC, SVC, and GBC for the overall survival prediction. Additionally, we also developed deep learning (DL) multilayer perceptron model.

#### Radiomic Feature Selection

We opted for a two-step process to select the best radiomic features for OS prediction out of 1093 radiomic features extracted from CT images. We selected 121 stable radiomic features based on our earlier radiomic stability study [21]. Subsequently, the top 50 features were selected using the Chi-squared test. Finally, the top 10 features were selected by applying recursive feature elimination (RFE) methods using random forest (RFE-RF). Python 3.9.0 software is used for the feature selection process.

#### Prediction Model Development and Validation

The prediction models were developed using random forest (RF), support vector (SV), and gradient boosting (GB) algorithms in Python 3.9.0 software. Hyperparameters of these prediction algorithms were tuned using nested cross-validation, and the same parameters were used to develop all the prediction models. Subsequently, 10-fold cross-validation was performed to access the model performance on the internal dataset. In the next step, a train-test split (80:20) was performed for model development and validation. Three prediction algorithms were used to develop a total of six prediction models utilizing original and balanced training sets.. RF models (RF-Model-O: on the original training data and RF-Model-B: on the balanced training data); SV models (SV-Model-O: on the original training data and SV-Model-B: on the balanced training data), and GB models (GB- Model-O: on the original training data and GB-Model-B: on the balanced training data) were developed on the internal dataset and validated on the test dataset. Subsequently, these models were also validated using the bootstrap (1000 iterations) method on the test dataset and on the external validation cohort.

Two deep learning models (simple-DL: 7-layer perceptron model without dropout layer and dropout-DL: 7-layer perceptron model with dropout layer) were also developed using an internal train-test dataset. Both the DL models were validated using the internal test dataset and an external dataset.

Using random forest, support vector, and gradient boosting algorithm, three models, i.e., RF-MODEL-V, SV-MODEL-V, and GB_MODELS-V, were also developed for predicting 2-year overall survival with tumor volume as a single feature.

#### Statistical Tests

For all the statistical tests, different packages of Python 3.9.0 open-source software were used. Descriptive statistical tests were performed to understand the distribution of patients in various categories. The demographic data of the internal and external cohorts were compared using *t*-test. Hierarchical clustering using Pearson’s correlation test and *z*-score and Chi-squared tests was performed for feature reduction. Recursive feature elimination using a random forest algorithm was performed to select the most significant features for model development. The features from both cohorts were compared using a *t*-test and violin plot. Receiver operating characteristics area under the curve (AUC), accuracy, precision, recall, and f1-score were calculated for all prediction models on internal and external validation datasets.

## Results

**Fig. 1:**
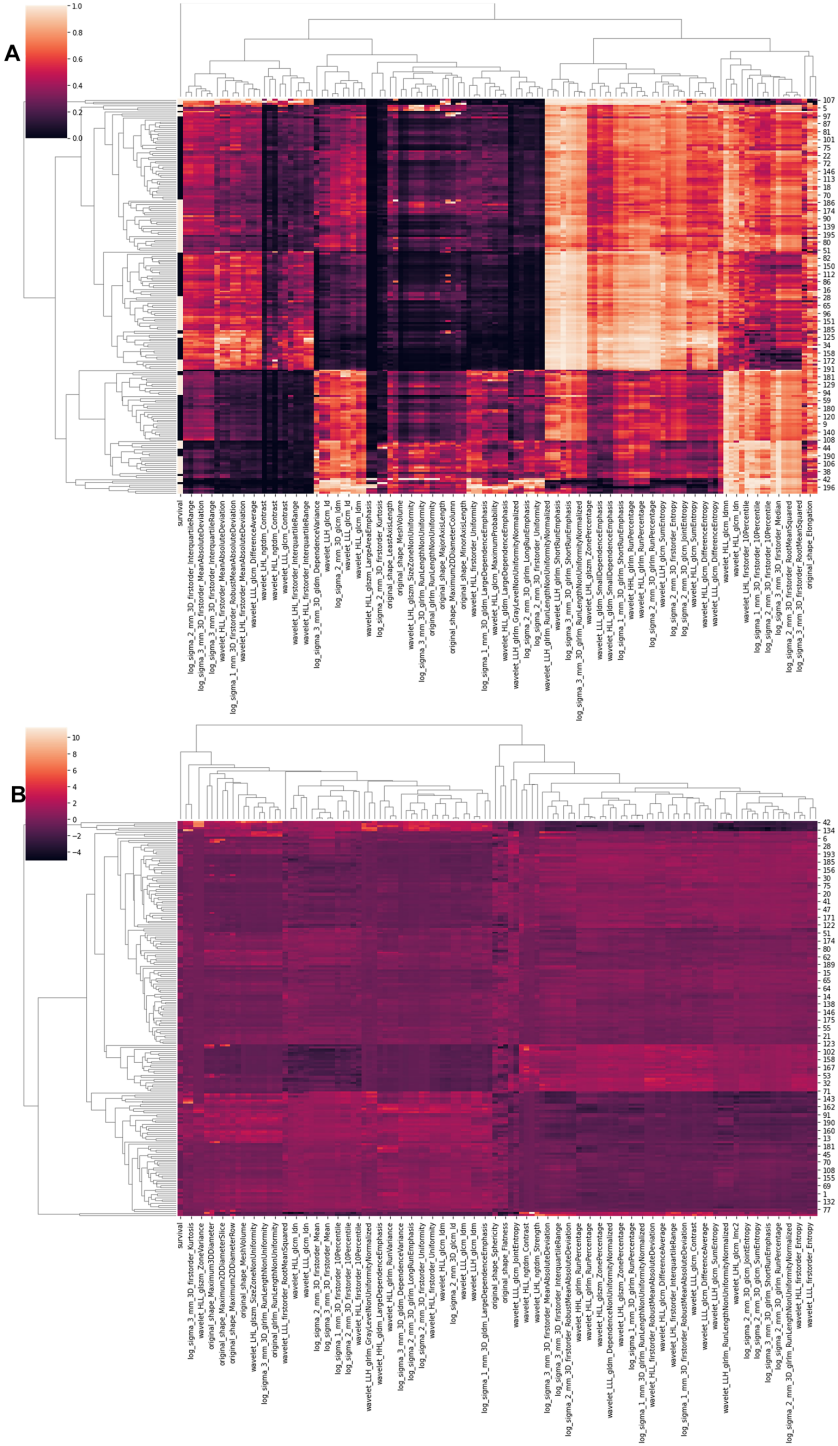
Heatmap of 121 radiomic features based on **A** Pearson’s correlation test and **B** *z*-score. Hierarchical clustering shows the clusters of radiomic features

**Fig. 2:**
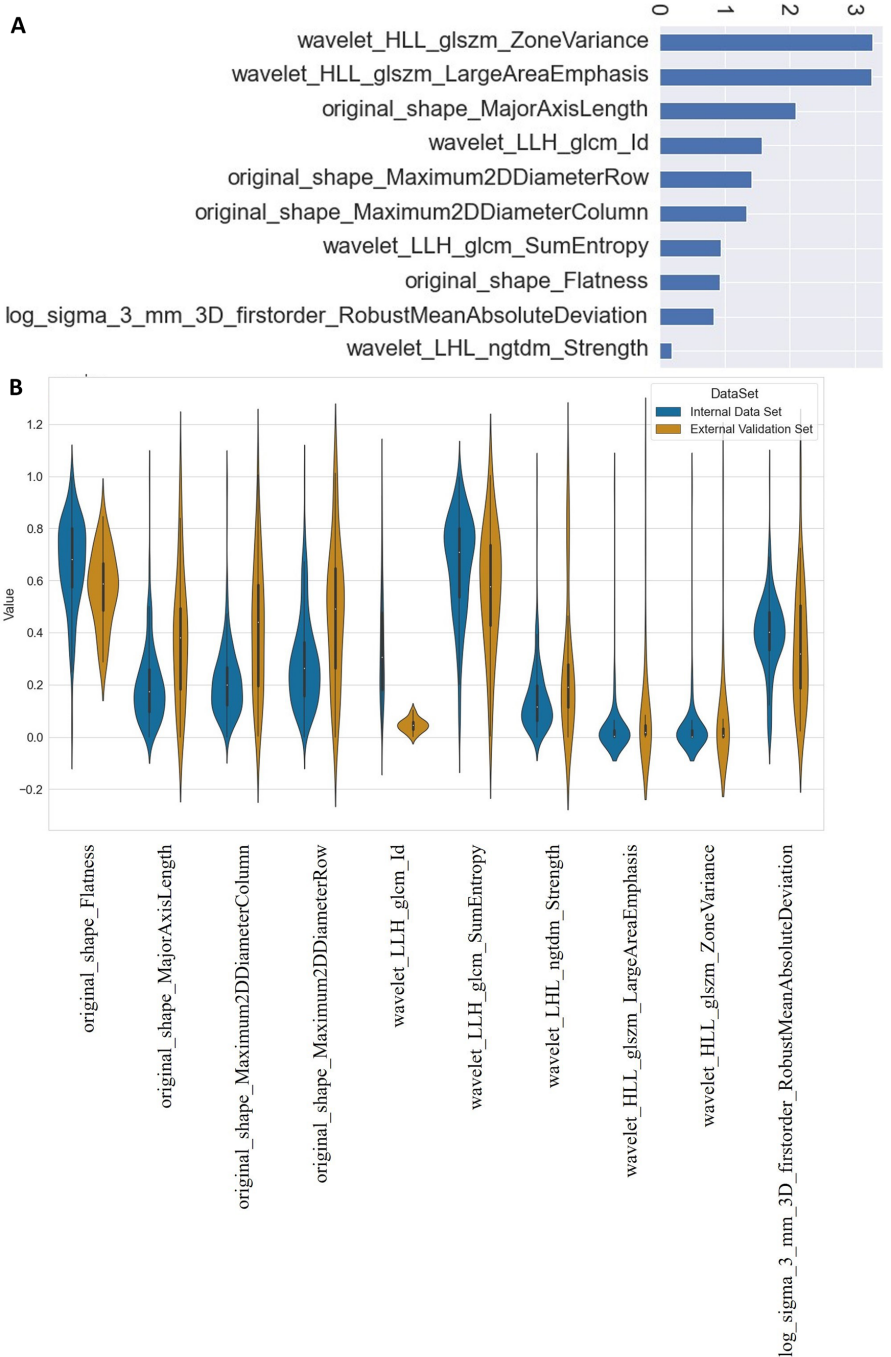
The feature significance of the ten most important features on the Chi-squared test (**A**), the distribution of min–max scaled feature values of the top 10 significant features for the TMH cohort and the validation cohort (**B**)

The descriptive statistics of demographic data and comparison for both cohorts are shown in Table 1. The heatmap with hierarchical clustering and* z*-score heatmap of 121 stable radiomic features shows several feature clusters (Fig. 1). Subsequently, based on hierarchical clustering and the multivariate Chi-squared test, top 50 significant features were selected. Finally, the RFE technique was applied using the random forest algorithm, and the 10 most significant radiomic features were selected for model development. The significance of 10 selected features on the internal dataset using the Chi-squared test and the comparison of the distribution feature values on internal and external datasets are shown in the bar chart (Fig. 2A) and violin plot (Fig. 2B), respectively. The comparison of ten significant radiomic features between internal and external cohorts is shown in Table 2. The violin plot and *t*-test show a similarity in feature distribution for the majority of selected features in internal and external datasets except for a few (Fig. 2).

**Table 2:** The results of the unpaired *t*-test showing the relation between the features of the two datasets

**Features**	***t*-statistics**	***p* value**
original_shape_Flatness	2.02	0.04
original_shape_MajorAxisLength	1.28	0.20
original_shape_Maximum2DDiameterColumn	−0.99	0.32
original_shape_Maximum2DDiameterRow	1.09	0.27
wavelet_LLH_glcm_Id	−2.74	0.006
wavelet_LLH_glcm_SumEntropy	−104.08	2.43e-236
wavelet_LHL_ngtdm_Strength	−10.89	1.75e-23
wavelet_HLL_glszm_LargeAreaEmphasis	1.66	0.097
wavelet_HLL_glszm_ZoneVariance	1.66	0.094
log_sigma_3_mm_3D_firstorder_RobustMeanAbsoluteDeviation	0.26	0.79

**Fig. 3:**
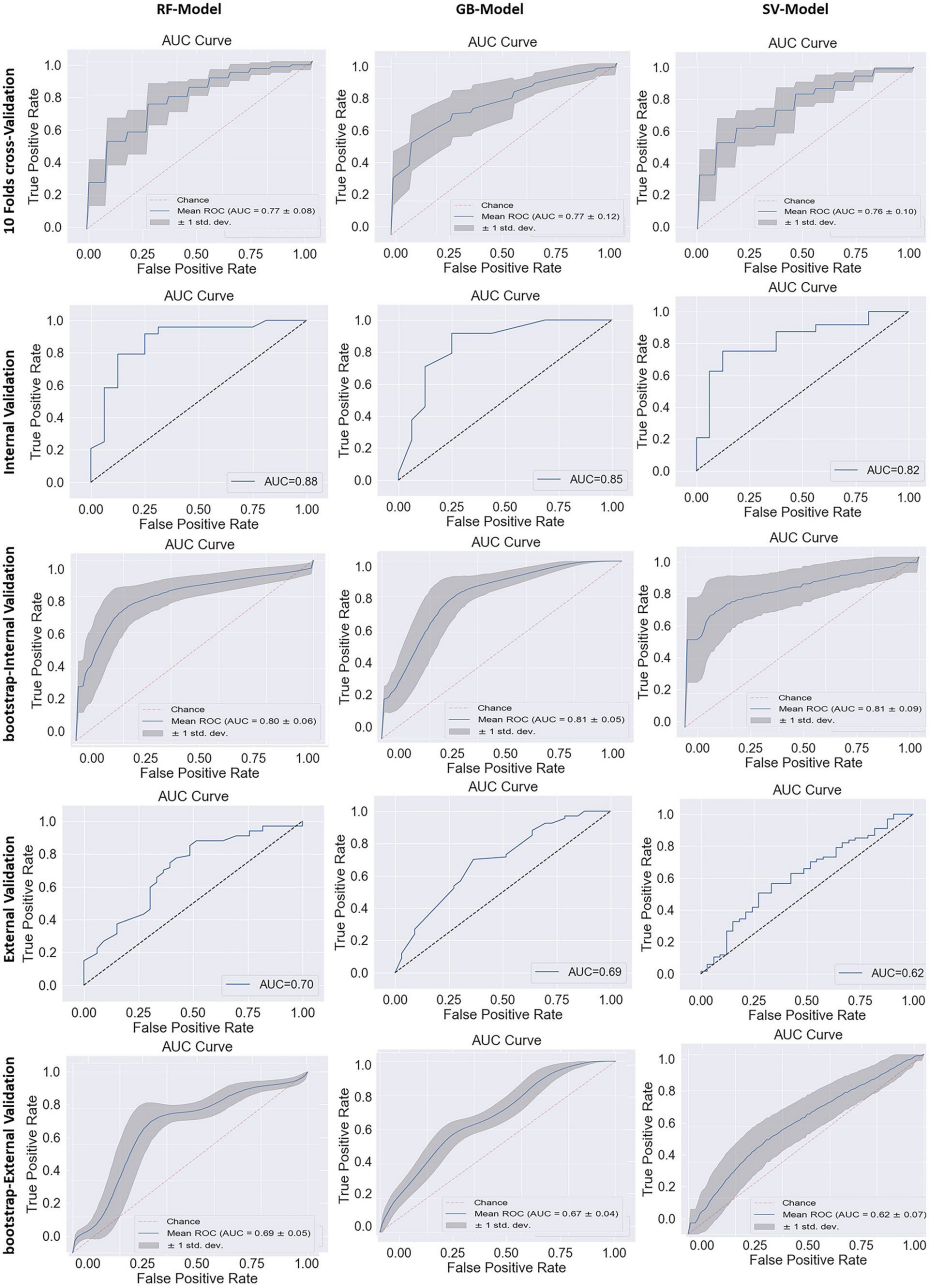
The ROC-AUC curve in 10-fold cross-validation, internal validation, and external validation. AUC curves of the random forest model, gradient boosting model, and support vector model are shown in the first, second, and last rows, respectively

**Fig. 4:**
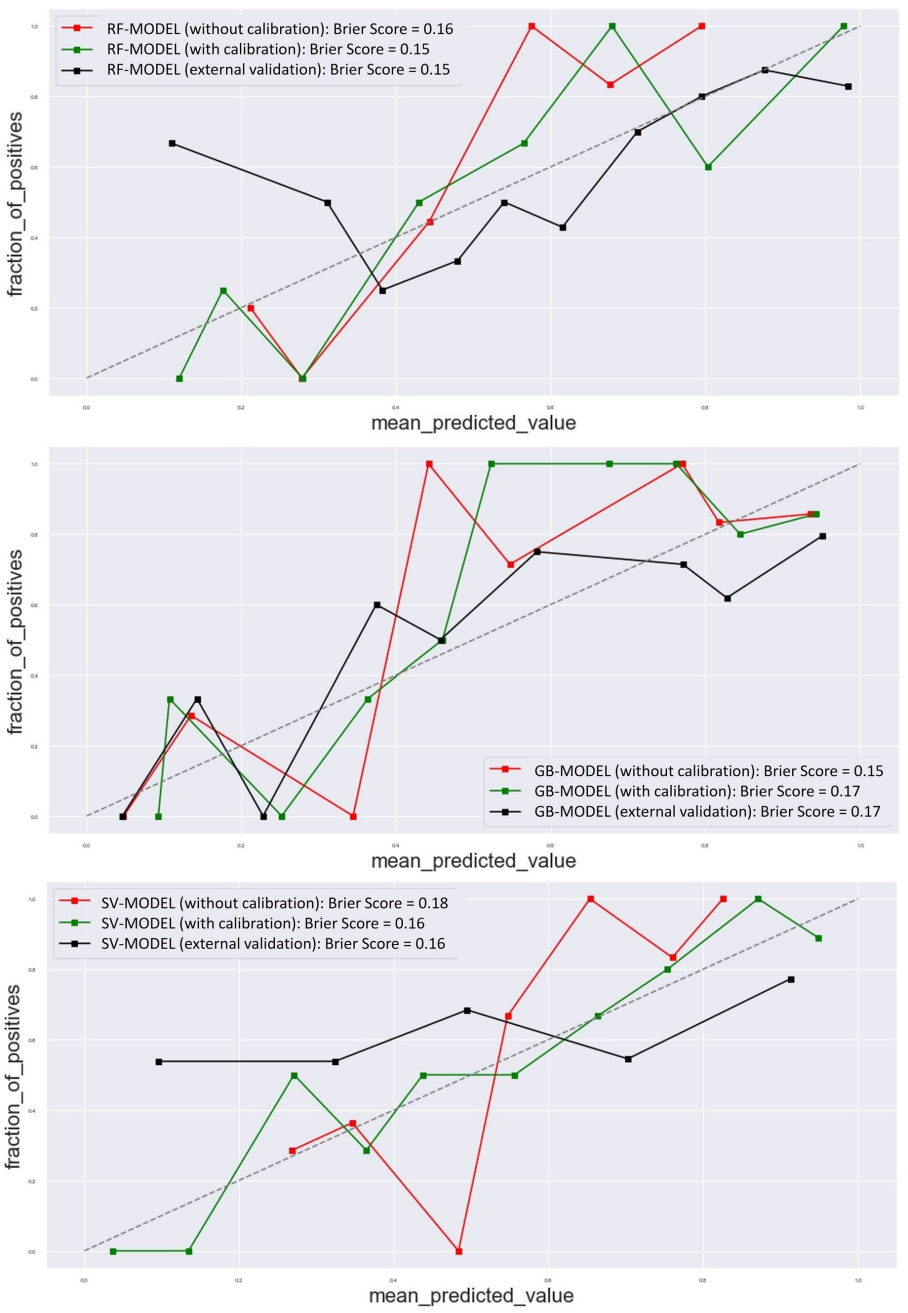
The calibration plot and Brier scores of the prediction models with and without calibration in the internal validation set. The calibration plot and Brier score of random forest model, gradient boosting model, and support vector model in the first, second, and last row, respectively

The 10-fold cross-validation on the institutional (TMH) dataset showed a good prediction accuracy and AUC of 0.73 ± 0.08 and 0.77 ± 0.08 for RF-Model-O, 0.69 ± 0.12 and 0.76 ± 0.09 for SV-Model-O, and 0.73 ± 0.00 and 0.79 ± 0.08 for GB-Model-O, respectively. The accuracy of models in train-test internal validation was between 0.76 (for SV-Model-O) to 0.80 (RF-Model-O/GB-Model-O) and AUC 0.81 (RF-Model-O/GB-Model-O) to 0.82 (SV-Model-O) with the original training set (Fig. 3). The training and test prediction scores for all the models developed on the original dataset were found to be the same (Supplementary Table s2) The comparison of prediction models developed using the original and balanced training set was found to be comparable (Table 3). The accuracy of the external validation cohort was found to be between 0.57 (SV-Model-O) and 0.68 (RF-Model-O/GB-Model-O) and AUC 0.61(SV-Model-O) to 0.72 (RF-Model-O/GB-Model-O) (Fig. 3). The test and external validation prediction scores were found to be comparable to that of the bootstrap validation respectively. The internal and external validation results for all the models are shown in Table 4. The post-calibration model accuracy in internal validation was also found to be the same. The ROC curve of all the models for cross-validation, internal validation, and external validation and bootstrap validation is shown in Fig. 3. The test ROC curve and confusion matrix for prediction models developed on original and balanced training sets are shown in Supplementary Figs. s1–s3. The test prediction scores for prediction models developed on original and balanced training sets are shown in Table 3. The detailed prediction scores of prediction models in internal and external validation are shown in Table 4. The confusion matrix of internal and external validation is shown in Supplementary Fig. 4. The calibration plots of all three models are shown in Fig. 4. The deep learning models (simple-DL and dropout-DL) also performed well with accuracy = 0.76 and AUC = 0.72, respectively. However, these models failed in external validation with an accuracy of 0.55 for both models. The detailed model performance scores are shown in Table 4.

**Table 3:** The comparison of the prediction model developed on the original and balance dataset

**Prediction model**		**Dataset**	**Accuracy**	**Precision**	**Recall**	**f1-score**	**AUC**
**Random forest model**	RF-Model-O	Internal validation	0.83	0.84	0.82	0.83	0.87
	RF-Model-B		0.80	0.80	0.80	0.80	0.87
	RF-Model-O	External validation	0.68	0.66	0.68	0.67	0.69
	RF-Model-B		0.71	0.69	0.71	0.69	0.69
**Support vector**	SV-Model-O	Internal validation	0.78	0.80	0.78	0.78	0.82
	SV-Model-B		0.75	0.76	0.75	0.75	0.83
	SV-Model-O	External validation	0.57	0.62	0.57	0.58	0.61
	SV-Model-B		0.61	0.62	0.61	0.61	0.61
**Gradient boost**	GB-Model-O	Internal validation	0.80	0.81	0.80	0.80	0.81
	GB-Model-B		0.80	0.82	0.80	0.80	0.86
	GB-Model-O	External validation	0.68	0.66	0.68	0.67	0.72
	GB-Model-B		0.65	0.63	0.65	0.64	.65

*O* stands for original data set, *B* stands for balanced dataset

**Table 4:** The performance of prediction models in internal and external validation

**Algorithm**	**Dataset**	**Model**	**Accuracy**	**Classification report**			**AUC**
				**Precision**	**Recall**	**f1-score**	
Random forest classifier (RFC)	10-Fold cross-validation	RFC	0.72 ± 0.10	–	–	–	0.77 ± 0.08
	Internal validation	RF-Model-O	0.83	0.84	0.82	0.83	0.87
	Bootstrap-internal validation	RF-Model-O	0.81 ± 0.05	0.83 ± 0.05	0.80 ± 0.07	0.82 ± 0.05	0.80 ± 0.06
	External validation	RF-Model-O	0.68	0.66	0.68	0.67	0.69
	Bootstrap-external validation	RF-Model-O	0.72 ± 0.04	0.77 ± 0.06	0.85 ± 0.03	0.81 ± 0.03	0.69 ± 0.05
Support vector classifier (SVC)	10-Fold cross-validation	SVC	0.69 ± 0.12	–	–	–	0.76 ± 0.09
	Internal validation	SV-Model-O	0.78	0.80	0.78	0.78	0.82
	Bootstrap-internal validation	SV-Model-O	0.78 ± 0.08	0.94 ± 0.07	0.68 ± 0.12	0.81 ± 0.03	0.81 ± 0.09
	External validation	SV-Model-O	0.57	0.62	0.57	0.58	0.61
	Bootstrap-external validation	SV-Model-O	0.57 ± 0.06	0.73 ± 0.07	0.57 ± 0.07	0.64 ± 0.06	0.61 ± 0.07
Gradient boosting classifier (GBC)	10-Fold cross-validation	GBC	0.73 ± 0.07	–	–	–	0.79 ± 0.08
	Internal validation	GB-Model-O	0.80	0.81	0.80	0.80	0.81
	Bootstrap-internal validation	GB-Model-O	0.72 ± 0.05	0.84 ± 0.06	0.67 ± 0.07	0.74 ± 0.06	0.81 ± 0.05
	External validation	GB-Model-O	0.68	0.66	0.68	0.67	0.72
	Bootstrap-external validation	GB-Model-O	0.70 ± 0.03	0.73 ± 0.04	0.86 ± 0.03	0.79 ± 0.03	0.67 ± 0.04
7-layer perceptron model	Internal validation	Simple-DL	0.76	0.7	0.73	0.71	0.72
		Dropout-DL	0.76	0.7	0.73	0.71	0.72
	External validation	Simple-DL	0.55				
		Dropout-DL	0.55

The accuracy of the tumor volume-based models, RF-Model-V, SV-Model-V, and GB-Model-V, in the internal validation set was found to be 0.57, 0.50, and 0.51, respectively. The details of the internal validation scores of these volume-based models are shown in the supplementary material (Supplementary Table s3 and Fig. s5).

## Discussion

In the last few years, radiomics has been a major area of research in oncology to develop digital phenotypes for various cancers [10–19]. Several radiomics-based prediction models have been developed, validated, and reported in the literature to predict various prediction endpoints in various cancer types. The role of radiomic features in the prediction of various clinical endpoints in lung cancer has been investigated and reported widely in the last few years [10–19, 27, 28, 30, 32–35]. But the generalization of these radiomic models has raised concern in the radiomics community. The high number of radiomic features extracted from the medical images of tumors leads to a data explosion. This data explosion raises several concerns like issues related to feature repeatability and reproducibility, feature redundancy, feature insignificance, and multidimensionality which is the biggest challenge facing the radiomic community. To overcome these challenges, the model development needs to undergo several steps, i.e., identification of stable features, removal of redundant features, selection of most significant features, and finally selection of the best prediction algorithm [29]. In this study, we have implemented a multistep feature selection process to identify the most suitable features for the overall survival prediction in NSCLC patients. Based on our earlier study on the repeatability and reproducibility of CT radiomic features, we selected 121 robust radiomic features out of 1093 extracted features [21] for this study to minimize the drawback related to the reproducibility of radiomic features. To reduce the redundancy, we used Chi-squared and hierarchical clustering using Pearson’s correlation and *z*-score analysis to identify and remove redundant features. Subsequently, we used a random forest algorithm-based recursive feature elimination (RFE) technique to identify the most suitable radiomic features to predict 2-year overall survival. In our investigation, these radiomic features were able to risk stratify patients into two groups and predict the overall survival in this cohort of patients. These radiomic features show a strong correlation with 2-year overall survival. We used the three most common machine learning algorithms, i.e., RFC, SVC, and GBC and the deep learning multilayer perceptron model to develop a prediction model. The average accuracy of RFC and GBC is similar with an accuracy of around 0.80 and has better accuracy than the SVC prediction model in internal validation. The training and test prediction scores for all the models were found to be comparable (Supplementary Table s2). Comparable training and test prediction scores indicate the reduced probability of model overfitting. Several studies in the past have reported similar findings and our results affirm these findings [26, 27]. Similarly, the fact that GBC and RFC models performed equally well on 10-fold cross-validation and bootstrap validation suggests a robust prediction model. In external validation, the accuracy of GBC and RFC models was also found to be comparable and equally good (accuracy > 0.70). The calibration plot of GBC and RFC also shows similarity in the internal and external validation with a Brier score of around 0.15. The slight reduction in prediction accuracy matrices may be attributed to differences in the two cohorts as shown in Table 1. Some features also have different distributions in two cohorts as shown in the *t*-test and violin plot. However, bootstrap validation on internal tests and external cohorts shows the stability of radiomic-based prediction model in NSCLC. The deep learning model also showed good accuracy around 0.72 in internal validation but failed miserably in external validation. Both the deep learning models performed equally well in internal validation, whereas these models failed miserably in external validation suggesting the overfitting of the models.

As our development and internal validation cohort include NSCLC patients of stages I–IV, it establishes the fact that this model can predict the event rate across the disease stage. The external validation cohort had several dissimilarities from that of the development cohort; i.e., it consists of stages I–III, the difference in the median age was around 13 years, the difference in median overall survival, and the difference in treatment offered. Nevertheless, the prediction model performed well, and this also strengthens the claim of the generalized nature of this prediction model.

There is concern among the radiomic community regarding the feature stability and predictability of the radiomic model in external validation. Several studies have been performed to address the issues related to the stability of radiomic features [21, 22]. In our earlier study, we performed a rigorous analysis on a human cohort and phantom study to identify the most robust radiomic features [21]. In this study, we were successfully able to demonstrate two aspects of a good radiomic feature: (1) the predictive power and (2) the stability of robust radiomics-based prediction model in external validation. The results of the prediction models on internal validation confirm the predictive potential of these robust radiomic features. We have chosen the top 10 radiomic features from 121 robust radiomic features that offered the highest accuracy in RFE using a random forest algorithm. While the success of these prediction models especially random forest and gradient boost models in external validation is encouraging, radiomic-based prediction models may be generalized if feature stability is thoroughly and accurately assessed. Le et al. in their study have shown the ability of radiomic features to predict the overall survival using the cox model. In this study, the authors have also selected ten significant radiomic features similar to our study for model development [35]. A similar study by the same author demonstrated the predictive power of the radiomic-based prediction model in discriminating the patient in high- and low-risk groups as well as the overall survival prediction in multiorgan cancer [36]. With our study, we have assessed and demonstrated the predictive capability of robust radiomic features in the prediction of 2 years of the overall survival in lung cancer, which was also validated on external datasets. However, the radiomic features selected in our study are different from that of Le et al. which may be because of the features included at the beginning of the feature selection step, feature selection techniques used, and prediction algorithms used.

Several studies in the past have suggested the role of decision support systems (DSS) in clinical decision-making [32–35]. Prediction of the overall survival is one of the important clinical questions in oncology that can be answered by a DSS. Our study can contribute significantly to the development of a DSS for the prediction of the overall survival in lung cancer.

The current study has a number of limitations, including its retrospective nature and limited sample size, as well as a heterogeneous cohort. To address the issue of small sample size, we employed the cross-validation and bootstrap validation approach for model validation. In future research, our goal is to validate this model on a multicentric study retrospectively. Subsequently, this model will be trained on the large retrospective dataset and validated on the prospective dataset from a multicentric study. The ultimate objective of this research is to validate this prediction model in multicentric prospective clinical trials and implementation of the decision support systems in clinics.

## Conclusion

Robust radiomic features have shown promising results for the prediction of 2-year overall survival in non-small cell lung cancer. Comparing the SVC model, the RFC and GBC models performed better in internal and external validation. Despite the fact that this is merely an early study on a small development and validation dataset, the results of external validation suggest that the radiomic-based prediction model may eventually be generalized.

### Supplementary Information

Below is the link to the electronic supplementary material.

Supplementary file1 (DOCX 547 KB)

## Data Availability

Data is available with the correspondence author. Data sharing is not permitted as per the IRB approval.
