# Supplementary material for: External Validation of Robust Radiomic Signature to Predict 2-Year Overall Survival in Non-Small-Cell Lung Cancer
Source: J Digit Imaging. 2023 Sep 21;36(6):2519–31. doi: 10.1007/s10278-023-00835-8 (PMC10584779; doi:10.1007/s10278-023-00835-8)
Supplement: Supplementary file 1 — Supplementary file1 (DOCX 547 KB) [file 10278_2023_835_MOESM1_ESM.docx]

External Validation of Robust Radiomic Signature to Predict 2-Year Overall survival Non-Small-Cell Lung Cancer

In this study, we have developed the prediction model using radiomics features and three prediction algorithms i.e. Random forest classifier, Gradient Boosting classifier and support vector classifier.

Pre-therapeutic Computed Tomography images are used for radiomic extraction. The imaging parameters of CT images are shown in table s1.

**Random Forest Classifier (RFC):** An ensemble machine learning approach called Random Forest classifier makes use of bagging or Bootstrap Aggregation. RF samples the data many times and builds a different prediction model for every sample. RF determines the genuine mean value of the model by averaging the outcomes of all the models. All of the models offered to predict both the ensemble outcome value and the individual outcome.

**Support Vector Classifier (SVC):** Support vector classifier constructs a classifier to establish a hyperplane, or decision border, between two classes of data. The nearest data points from each of the data classes that this hyperplane is oriented in are known as support vectors. Using SVM, we can model larger dimensional, non-linear models, which is highly helpful.

**Gradient Boosting Classifier (GBC):** A group of machine learning techniques called gradient boosting classifiers combine several weak learning models to create a potent predicting model. Decision trees are frequently employed in gradient boosting. Gradient boosting classification techniques typically minimize the loss function, or the difference between the actual class and the predicted class, using a logarithmic loss function.

**The recursive feature elimination (RFE)** technique was used to select the most relevant features for predicting 2-year overall survival. In total 10 radiomic features were selected to predict 2-year overall survival.

Model performance was assessed on the internal and external validation sets (The cancer Image Archive (TCIA)). Several studies suggest the high prediction power of tumor volume hence the performance of the prediction models developed in this study was also compared to the models developed using the same prediction algorithms and tumor volume as a single feature (table s2).

| Cohort | Tube Voltage (kVp) | Tube current (mA) | Slice thickness (mm) | Pitch (mm) | pixel spacing  (mm) | Reconstruction Technique |
| --- | --- | --- | --- | --- | --- | --- |
| TMH | 120 | 100-200 Auto-mA | 3.75 | 3.75 | 1.17 | Filtered back project (FBP) |
| Lung-1 | 120 | 200-400 mA | 5 | 1.5 | 0.7- 1.0 | Filtered back project (FBP) |

Table S 1: Overview of CT imaging protocol

Table 2s shows the prediction scores i.e., accuracy, precision, recall, f1-score and ROC AUC of all three original models. The training and test prediction scores were found to be similar.

| Prediction Model | Dataset | Accuracy | Precision | Recall | f1-score | AUC |
| --- | --- | --- | --- | --- | --- | --- |
| RF-Model-O | Train | 0.81 | 0.80 | 0.80 | 0.80 | 0.82 |
|  | Test | 0.83 | 0.84 | 0.82 | 0.83 | 0.87 |
| SV-Model-O | Train | 0.73 | 0.76 | 0.73 | 0.71 | 0.78 |
|  | Test | 0.78 | 0.80 | 0.78 | 0.78 | 0.82 |
| GB-Model-O | Train | 0.83 | 0.83 | 0.82 | 0.82 | 0.83 |
|  | Test | 0.80 | 0.81 | 0.80 | 0.80 | 0.81 |

Table S 2: Training and test prediction scores for various prediction models

The prediction models developed on the original and balanced training were found to be comparable based on the test and external validation prediction scores (figure s1-s3).


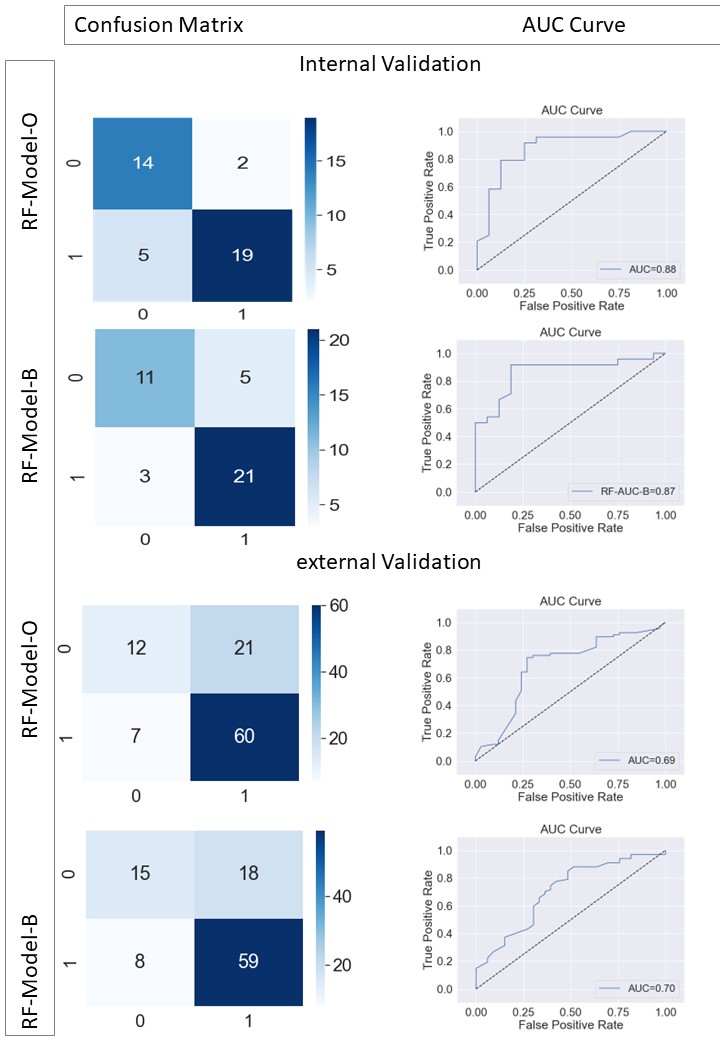


Figure s1: Figure shows prediction performance (confusion matrix) of random forest model developed on original and balanced dataset.


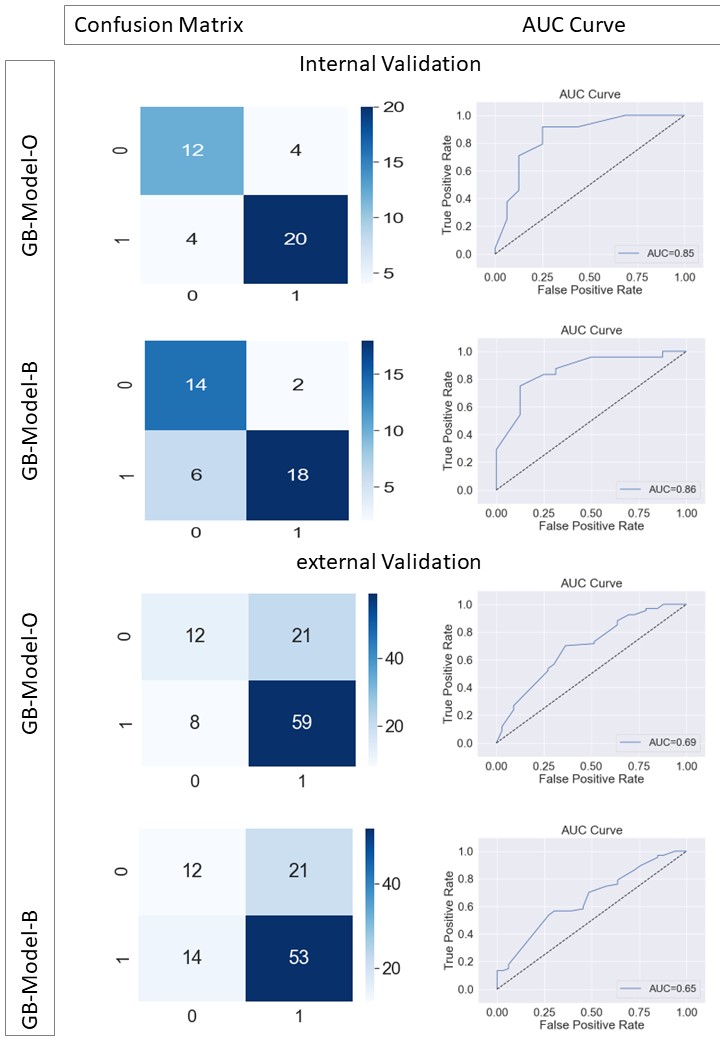


Figure s2: Figure shows the prediction performance (confusion matrix and ROC curve) of the Gradient boosting model developed on the original and balanced dataset.


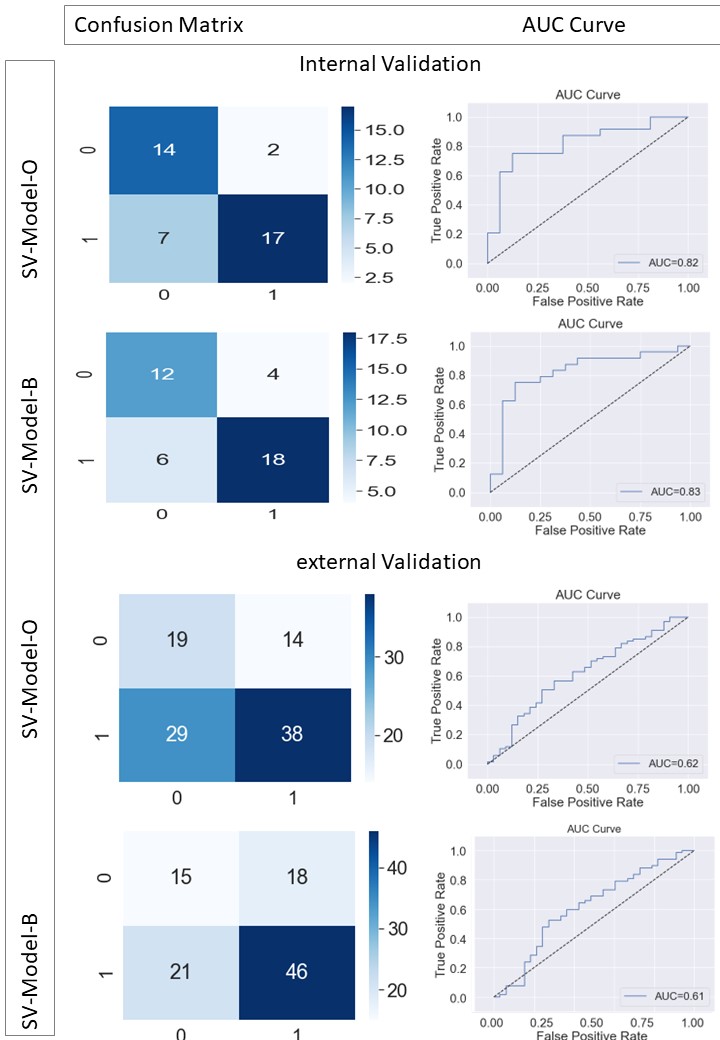
Figure s3: Figure shows prediction performance (confusion matrix and ROC curve) of Support vector model developed on original and balanced dataset.


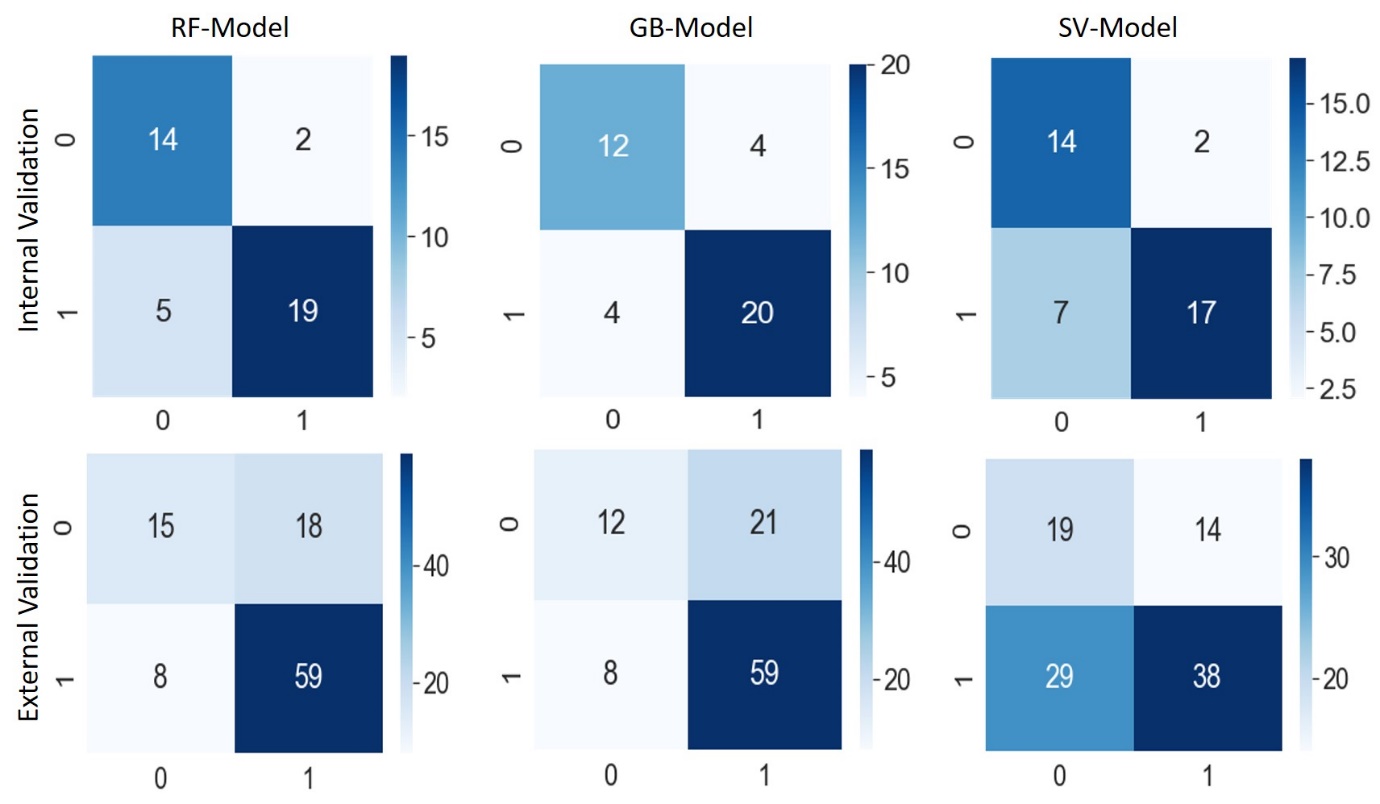


Figure S4: The figure shows the confusion matrix of the performance of the radiomic prediction models in the internal and external validation set.

| **Model** | **Accuracy** | **Classification report** | | | **AUC** |
| --- | --- | --- | --- | --- | --- |
|  |  | **Precision** | **Recall** | **f1-score** |  |
| RF-Model-V | 0.57 | 0.55 | 0.56 | 0.55 | 0.57 |
| GB-Model-V | 0.53 | 0.47 | 0.47 | 0.47 | 0.50 |
| SV-Model-V | 0.5 | 0.43 | 0.43 | 0.43 | 0.49 |

Table S 3: The table shows the prediction performance of tumour volume-based model in the internal validation set


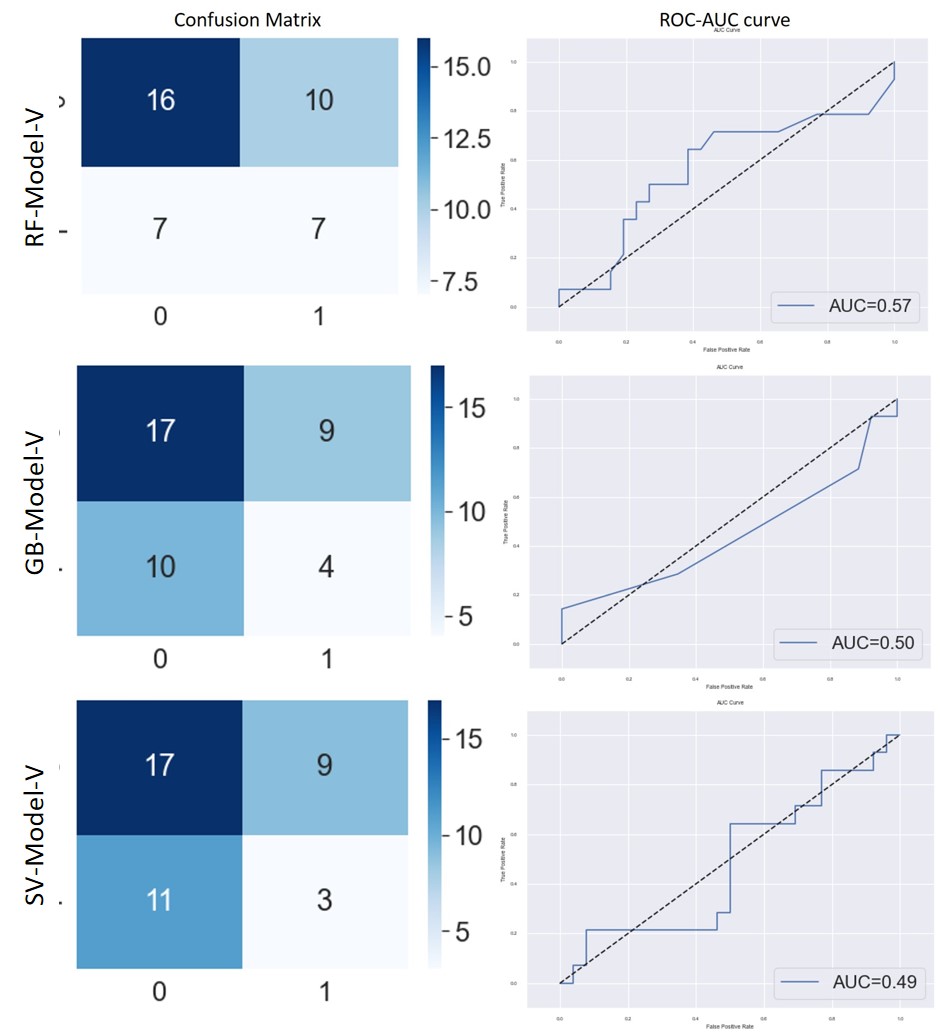


Figure S 5: The figure shows the confusion matrix and ROC-AUC curve of the prediction models developed using tumour volume in internal validation. The first row shows the prediction results (confusion matrix and ROC-AUC curve) of the random forest model, the second row shows the performance of the Gradient boosting model and the last row shows the performance of the support vector model developed using volume.
